# Draft Genome of the Heterotrophic Iron-Oxidizing Bacterium “*Acidibacillus ferroxidans*” Huett2, Isolated from a Mine Drainage Ditch in Freiberg, Germany

**DOI:** 10.1128/genomeA.00323-17

**Published:** 2017-05-11

**Authors:** Simone Schopf, Sophie R. Ullrich, Thomas Heine, Michael Schlömann

**Affiliations:** Institute for Biosciences, Environmental Microbiology, TU Bergakademie Freiberg, Freiberg, Germany

## Abstract

Here, we communicate the draft genome of “*Acidibacillus ferrooxidans*” Huett2, a novel strain of an acidophilic, heterotrophic, iron-oxidizing bacterium belonging to the phylum* Firmicutes*. It was isolated from a water drainage system of a former mine field in Freiberg, Germany.

## GENOME ANNOUNCEMENT

Recently, the isolation and characterization of novel acidophilic iron and sulfur oxidizing bacteria from mining-affected locations and geothermal sites all over the world were reported (1). The Gram-positive isolates belong to the newly described genus “*Acidibacillus*” within the *Firmicutes*. All characterized strains of “*Acidibacillus ferrooxidans*” (mesophilic iron oxidizers) and “*Acidibacillus sulfuroxidans*” (moderately thermophilic iron- and sulfur oxidizers) are strictly dependent on a source of organic carbon. The region of Freiberg within the ore mountains in Germany is marked by centuries of mining activities. The novel *A. ferrooxidans* strain Huett2 was isolated from water of the mine drainage ditch “Hüttenrösche” which was streaked out on solid plates supporting the growth of acidophilic bacteria. Hereinafter, the isolate was cultivated in liquid MAC medium (2) with pH 2.3, 25 mM ferrous iron, and 0.05% yeast extract. *A. ferrooxidans* Huett2 oxidized ferrous iron but not elemental sulfur and did not grow without yeast extract. It catalyzed the oxidative dissolution of pyrite and sphalerite by bioleaching.

The genome of *A. ferrooxidans* Huett2 was sequenced using an in-house MiSeq sequencing machine (Illumina, San Diego, CA, USA). The library was prepared with the NexteraXT DNA library kit (Illumina, San Diego, CA, USA) and checked with an Agilent technology bioanalyzer 2100. Paired-end libraries were sequenced using Illumina v3 chemistry. The resulting 8,040,162 reads were uploaded to the web-based genome analysis tool Galaxy (http://galaxy.uni-freiburg.de [3–5]). Read quality was assessed using FastQC. Reads were quality trimmed with TrimGalore! applying quality filter Phred20. A *de novo* assembly was generated based on 8,000,616 trimmed reads (approximately 660-fold genome average) using SPAdes (6). Small contigs (<1,000 bp) and those with low recovery (<10) were removed from the assembly. Quality assessment of the genome assembly was with QUAST (7). The final assembly produced 50 contigs with a size of 2.99 MB, a *N*_50_ of 176,325, and an estimated G+C content of 52.2%. The DNA assembly was submitted to the NCBI Prokaryotic Genome Annotation Pipeline for functional annotation of 2,883 protein coding genes. One hundred eight pseudogenes, 58 RNAs, and 1 clustered regularly interspaced short palindromic repeat (CRISPR) array were predicted.

### Accession number(s).

This whole-genome shotgun project has been deposited at GenBank under the accession number MWPS00000000.
